# Genetic and environmental influences on human dental variation: A critical evaluation of studies involving twins^☆^

**DOI:** 10.1016/j.archoralbio.2008.06.009

**Published:** 2009-12

**Authors:** Grant Townsend, Toby Hughes, Michelle Luciano, Michelle Bockmann, Alan Brook

**Affiliations:** a School of Dentistry, University of Adelaide, Australia; b School of Philosophy, Psychology and Language Studies, University of Edinburgh, United Kingdom; c International Collaborating Centre in Oro-facial Genetics and Development, University of Liverpool, United Kingdom

**Keywords:** Twin studies, Dental variation, Model fitting

## Abstract

Utilising data derived from twins and their families, different approaches can be applied to study genetic and environmental influences on human dental variation. The different methods have advantages and limitations and special features of the twinning process are important to consider. Model-fitting approaches have shown that different combinations of additive genetic variance (A), non-additive genetic variance (D), common environmental variance (C), and unique environmental variance (E) contribute to phenotypic variation within the dentition, reflecting different ontogenetic and phylogenetic influences. Epigenetic factors are also proposed as important in explaining differences in the dentitions of monozygotic co-twins. Heritability estimates are high for most tooth size variables, for Carabelli trait and for dental arch dimensions, moderate for intercuspal distances, and low for some occlusal traits. In addition to estimating the contributions of unmeasured genetic and environmental influences to phenotypic variation, structural equation models can also be used to test the effects of measured genetic and environmental factors. Whole-genome linkage analysis, association analysis of putative candidate genes, and whole genome association approaches, now offer exciting opportunities to locate key genes involved in human dental development.

## 1 The classical twin model and its assumptions

Monozygotic (MZ) co-twins share the same genes, whereas dizygotic (DZ) co-twins on average share only half of their genes. Therefore, by assuming that both types of twins have been sampled from the same gene pool and that similar environmental factors act upon them, one can estimate the relative contributions of genetic and environmental influences to observed variation in different features or traits. The calculation of heritability estimates provides a means of quantifying the extent of the genetic contribution to phenotypic variation, with proportions ranging theoretically from 0 to 1. Various formulae can be utilised to calculate estimates of heritability for both quantitative and categorical data, and their standard errors, although few early twin studies provided such estimates. Two types of heritability can be distinguished: ‘narrow-sense’ heritability refers to the contribution of additive genetic variance to observed phenotypic variance, whereas ‘broad-sense’ heritability refers to the total contribution of genetic factors (additive and non-additive) to the observed variation. Additive effects represent the sum of parental genes influencing the offspring's trait, whereas non-additive effects encompass the effects of genetic dominance and gene–gene interaction.

There are several assumptions that underlie the classical twin approach and these were not tested fully in many of the early studies. Furthermore, it has often been overlooked that heritability is a population concept, referring to the proportion of genetic variation within a given population at a particular time. The concept should not be applied to a single individual but, rather, to a group of individuals.^1^ In addition, as Smith and Bailit^2^ have pointed out, “contrary to popular opinion, the extent to which genes determine a trait has no relationship whatsoever with the success of environmental intervention”.

Kang et al.^3^ and Christian^4^ have outlined some of the assumptions that are implicit in using the classical twin model to partition variance into genetic and environmental components. The mean values for the trait under investigation should not differ between zygosity groups. Total variance within zygosities should also be equal for the model to hold, as heterogeneity of total variance suggests that environmental factors are not equal for MZ and DZ twins. Environmental covariances should also be equal, with heritability estimates being inflated if environmental covariance is greater in MZ twins than DZ twins. All of these assumptions should be tested statistically prior to calculating genetic and environmental contributions to phenotypic variance. Interestingly, Harris^5^ has recently noted heterogeneity in total variances for human odontometric data derived from twins, with DZ twins showing significantly larger within-pair values than MZ twins, leading him to question whether twins are representative of the broader population.

## 2 Criticisms of the twin model

A major issue of concern in many previous studies of twins has been the accuracy of zygosity determination. Although comparisons of physical appearance can provide a reasonably reliable means of determining zygosity, errors can occur and these may influence subsequent analyses. The use of blood groups, as well as serum and enzyme polymorphisms, improved the ability to assign zygosities to twins. More recently, the use of highly polymorphic regions of DNA derived from blood or buccal cells has proved to be accurate and reliable.^6^

One of the main criticisms of the classical twin model has been based on the assertion that MZ co-twins are likely to share more similar environments post-natally than DZ co-twins, so greater similarities between them compared with DZ co-twins may partly reflect more similar environments rather than more similar genetic constitutions. While this can be an important issue with some behavioural phenotypes, it is less likely to be a major factor in studies of dental morphology, although nutritional similarities could possibly affect dental development.

Another consideration is the possibility of an interaction between genetic and environmental influences. The classical twin model tends to assume that these two influences operate independently, hence the often-used phrase ‘nature versus nurture’. This is seldom the situation and there is frequent interaction between genetic and environmental factors.

A further criticism of the classical twin model has been whether it is reasonable to extrapolate the findings from twin studies to a general population containing many singletons, given the special nature of the twinning event, twin pregnancies and births, and the upbringing of twins. The nature of the phenotype under investigation is important when attempting to assess the importance of these factors. However, there are some who question whether this is an appropriate assumption, even for dental variables.^5,7^

## 3 Special features of the twinning process

The twinning process itself and the circumstances surrounding the birth of twins and their peri-natal development is special. Twinning has been associated with a high peri-natal mortality rate^8^ and MZ twins display a higher prevalence of congenital abnormalities, many of which appear to be related to failure of bilateral structures to fuse properly during development.^9^ Although some claim that the potentially harmful effects of twin gestation have been exaggerated,^10^ a large percentage of twins may not develop past 16 weeks post-conception, leading some researchers to refer to a ‘vanishing twin’ syndrome.^11^

Apart from an apparently higher prevalence of peri-natal mortality and morbidity amongst twins, there is another special feature of the twinning process that frequently has been overlooked. MZ twin pairs most often share a common placenta and chorion (around 60–70%), but there are around 20–30% of MZ co-twins who have separate placentas and chorions. Di-chorionic twins are thought to have separated at an early stage of development, probably in the first 5 days post-conception whereas mono-chorionic twins are thought to have separated at a later stage, around six to 9 days post-conception. In around 30% of mono-chorionic MZ twins, there can be arterio–venous anastomoses that can lead to marked differences in physical development. Few studies of dental features in twins have taken account of chorion type, although Burris and Harris^12,13^ have provided evidence that chorion type can affect permanent tooth dimensions. These researchers have suggested that previous estimates of heritabilities for dental traits, where these types of effects have not been considered, are likely to have been biased. In a recent study involving Australian twins, it was found that intrapair variances for tooth-size data in mono-chorionic twin pairs generally exceeded those for di-chorionic pairs, indicating that the prenatal environment of twins may have an effect on their developing dentitions.^14^

The fascinating phenomenon of mirror-imaging, where one member of a twin pair ‘mirrors’ the other for one or more features, is well known to most people. However, most of the studies of mirror-imaging in twins have been retrospective reports based on small sample sizes rather than being well-planned prospective studies. To ensure that findings are not purely due to chance, a suite of study variables needs to be defined, measurements and observations made, error studies performed, and comparisons of the frequencies of mirrored features made between MZ twins, DZ twins and singletons. Given that there is some preliminary evidence that mirror-imaging may be related to the timing of the division and therefore the type of placentation,^15^ information on chorion type of MZ twins would also be extremely valuable in any future study of mirror-imaging in twins.

## 4 Other twin models

Apart from the classical twin model, there are several other research methods that can be used when studying twins. One approach that overcomes the problem of possible confounding effects due to common family environment is to study MZ twins who were separated shortly after birth and subsequently reared in separate homes. Assuming that their adoption placement is not influenced by trait-related environmental factors, the similarity of reared apart MZ co-twins can be attributed to shared genes alone.^16^ Although these types of twins are rare, the Minnesota Study of Reared Apart Twins has enrolled more than 100 pairs of twins globally who were separated shortly after birth and then reunited in adulthood.^17,18^ Boraas et al.^19^ took advantage of this powerful approach to demonstrate a significant genetic contribution to permanent incisor tooth size, as well as to dental caries.

A further design is the MZ co-twin model, which involves comparison of MZ twins where each member of a pair has been exposed to different environmental effects. For example, MZ co-twins may be treated with different orthodontic appliances and the outcomes compared, or the severity of periodontal disease might be compared between MZ co-twins where one is a smoker and the other is not. The MZ co-twin model can also be used to make inferences about the relative contributions of genetic and environmental factors to phenotypes for which the twins are discordant. Differences in the number and position of missing teeth between MZ co-twins raise the likelihood that environmental and/or epigenetic influences during development can lead to quite distinct differences in dental development.^20^

Another research design involving twins is the so-called twin half-sib model.^21^ This approach involves studying genetic and environmental contributions to variation in MZ twins, their spouses and their offspring. Its advantage lies in the fact that the children of MZ co-twins who are born to different mothers are themselves genetically half-siblings. This method also offers the opportunity to detect maternal effects and to assess the importance of assortative mating, as multiple associations between MZ twins and their spouses can be explored.

Another model that can be used is the opposite-sexed DZ model. This approach focuses on male–female twin pairs and tests whether there are differences in mean values and variances for selected features between these twins compared with other twin types and singletons. Since each member of a male–female twin pair may be exposed to elevated levels of hormones from their co-twin in utero, it is possible that this may lead to observable effects post-natally. Indeed, there is some evidence that tooth size is increased in females belonging to opposite-sexed twin pairs compared with females from same-sexed MZ or DZ groups.^22^ It remains to be confirmed whether these apparent effects are due to the influence of male hormones on the female twin in utero, although other species provide some supportive evidence.^23^

## 5 Genetic analyses of dental variation using twin data

There have been two main quantitative genetic approaches used to clarify causes of observed variation in the human dentition: classical correlation analysis and multiple abstract variance analysis.^24^ The classical correlation approach compares the degree of association for selected traits between pairs of related individuals, with maximum correlation values that are assumed to be 1.0 for MZ co-twins and 0.5 for DZ co-twins. Estimates of heritability (h^2^) can be derived according to the formula: h^2^ = 2(rmz − rdz), where rmz and rdz are the values of correlation coefficients between samples of MZ and DZ co-twins for the feature under investigation.^25^ Variance analysis is a more systematic and comprehensive approach that compares within and between-family variances, leading to estimates of both genetic and environmental contributions to observed variation.^26^ A third method for analysing quantitative data is Fisher's biometrical approach that has several advantages over those previously described.^26^ Fisher's approach represented a major breakthrough in genetic analyses, as it enabled testing for components of variation, such as shared or common environmental factors, that were previously assumed to be absent or undetectable.

The development of more sophisticated model-fitting methods to analyse twin data made it possible to estimate the strength of genetic and environmental contributions within calculable confidence intervals.^27^ For example, the twin method can be used to estimate the relative influences of additive genetic factors (A), non-additive genetic factors (D), common or shared environmental factors (C) and environmental factors that are unique to the individual, including measurement error (E).

Three additional sources of variation and covariation between twins that need to be considered are assortative mating, genotype-by-environment interaction (GxE) and genotype–environment correlation (CorGE). The presence of assortative mating, if unmeasured, will lower the estimated genetic contribution to variation. To test for assortative mating, data from parents of twins are needed, as described previously in the twin half-sib model. It is unlikely that there is strong positive or negative assortative mating with respect to dental features, and the few attempts to explore this source of variation have failed to find evidence for its presence.^28^ G×E interaction refers to the situation in which one genotype may be expressed in the same way in two different environments but another genotype is expressed differently. This source of variation can be tested for by carrying out a regression analysis of MZ pair variances on MZ pair mean values for each study variable.^26^ CorGE occurs when the environments experienced by an individual are not a random sample of all environments but are correlated with the individual's genotype. There are several types of CorGE that act to increase or decrease estimates of genetic variance. One type of CorGE that may be relevant to studies of twins is a sibling effect where the phenotype of one sibling influences the phenotype of the other. Although little evidence that CorGE plays a significant role in twin studies has been reported,^10^ it should not be overlooked.

Computer programs, such as Mx developed by Neale,^29^ now facilitate simultaneous modelling of mean values, variances and covariances derived from twin data to be performed. Using Mx to apply sophisticated modelling methods to multivariate and longitudinal sets of dental data from twins offers great potential for clarifying how genetic and environmental factors influence development of the human dentition.

A summary of findings relating to genetic and environmental contributions to human dental variation based on the Adelaide twin studies is provided in Table 1.^24,30–33^ Structural equation models incorporating additive genetic variance (A) and unique environment variance (E) provide the best fits for several traits, including primary incisor emergence. Models that include only environmental variance, either unique environment (E) alone or a combination of common and unique environment (CE) provide the best fits for two molar intercuspal distances, emphasising the importance of non-genetic factors in determining variation in cusp position. Although AE models have been shown to provide the best fit for most permanent crown diameters, including maxillary central incisors, Table 1 shows two exceptions. Models including A, C and E components provide the best fit to explain variation observed in permanent first molar crown diameters, presumably reflecting the fact that these teeth commence calcification around birth. The ADE models provided the best fit for the mesiodistal diameters of maxillary canines. Genes related to selective fitness tend to show non-additive genetic variation, so the presence of this type of variation for canine size may reflect selective pressures operating on these teeth in particular, either currently or in the past. Heritability estimates for those features that displayed significant genetic variance differed considerably, with estimates for incisor and canine crown diameters and arch dimensions being relatively high, while those for intercuspal distances and molar crown diameters were moderate. Heritability estimates for occlusal traits, particularly incisor overjet, were low, highlighting a strong environmental contribution to observed variation. Model-fitting approaches have also been applied to dental data from Belgian twins, confirming a significant genetic contribution to variation in dental maturation with an ACE model providing the best fit.^34^

## 6 Focussing on the differences between MZ co-twins rather than the similarities

Our previous studies support the view that, even though there is a relatively strong genetic basis to missing or extra teeth, the number or position of affected teeth can be influenced by epigenetic factors.^20,35^ Although the precise nature of these influences is still unclear, they may be due to factors other than differences in methylation of DNA or acetylation of histones. We propose that they may reflect different responses of odontogenic cells to minor variations in the spatial and temporal expression of local signalling molecules passing between cells during development. In other words, we suggest that minor “disturbances” in epigenetic events at the local level during tooth formation can lead to quite major differences in the final appearance of the dentitions of MZ co-twins.

**Fig. 1:**
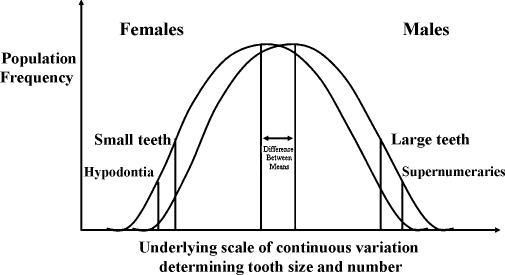
Multifactorial model with superimposed thresholds that explains the relationship between tooth size and missing or extra teeth in males and females. The figure is based on one presented originally by Brook (1984).

**Fig. 2:**
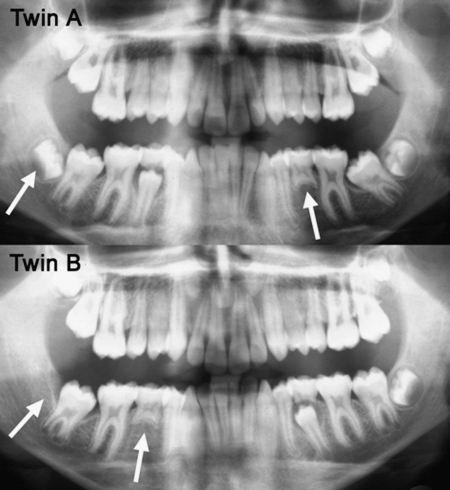
MZ co-twins showing mirror-imaging for missing lower second premolars and different expressions of third molar development, emphasising the role of epigenetic influences on dental development.

We consider that a multifactorial model^36^ – with genetic, epigenetic and environmental influences – provides the best explanation for our observations involving differences of expression of hypodontia and supernumerary teeth in MZ twin pairs (Fig. 1). Such a model, with superimposed thresholds linking tooth size, morphology and number, enables us to explain why MZ co-twins, who have the same genotypes, may display different expressions of missing, tapering and microdont incisors. Presumably these MZ twin pairs have a genetic predisposition for hypodontia that places them near the threshold for agenesis. However, minor variations in local epigenetic events during odontogenesis may lead to different phenotypic expression of lateral incisors between co-twins. A similar explanation may also account for the discordant patterns of missing premolars or supernumerary teeth within MZ co-twins. Presumably, these MZ twin pairs have a genetic make-up that places them near to a threshold for either missing or extra teeth, but variations in local epigenetic events during odontogenesis, probably relating to the spatial arrangement of cells or temporal events, determine on which side of the threshold they fall (Fig. 2).

Given that there is a link between the size and shape of teeth, and hypodontia or supernumerary teeth, we propose that there is likely to be a group of genes that exert pleiotropic effects on all of these dental phenotypes, accounting for their observed co-variation. How many genes are involved remains to be determined and it is possible that it may be a relatively small number. Support for this view is provided by Kangas et al.^37^ showing that dental characters seem to be non-independent and that increasing the levels of expression of just one gene can lead to increases in cusp number, altered cusp shape and position, development of longitudinal crests on teeth, and increases in tooth number in experimental mice.

## 7 Finding key genes

In addition to estimating the effects of unmeasured genes and environmental influences, structural equation models can also be used to test the effects of measured genetic and environmental factors. Until recently, the analysis of molecular genetic data focussed on two different approaches, namely whole-genome linkage analysis or association analysis of putative candidate genes, or a combination of the two. More recently the Wellcome Trust Case Control Consortium^38^ has applied whole genome association approaches as the method of choice. All three methodologies have distinct advantages and disadvantages, not the least of which is cost. The principles behind each are briefly summarised below.

### 7.1 Genome-wide linkage

Linkage analysis establishes relationships between the level of similarity in a phenotype in genetically related individuals and their levels of similarity in regions of the genome. If such a relationship can be established with sufficient statistical confidence, then one or more genes in those regions are possibly involved in phenotype similarity among individuals.

Linkage analysis depends on the co-segregation of alleles at a trait locus and a marker (i.e. on the same chromosome and thus violating Mendel's law of independent assortment). Markers are most commonly variable number tandem repeats – short segments of DNA with a repeated sequence, also called ‘microsatellites’. If a pair of offspring has received the same haplotype from a parent in a certain region of the genome, the pair is said to share that parent's alleles in that region identical by descent (IBD). Since offspring receive their haplotypes from two parents, the pair can share 0, 1 or 2 alleles IBD at a certain locus in the region. The IBD status is usually estimated for a number of markers with (approximately) known location along the genome (e.g. markers evenly spaced at 4 cM (1000 markers total) or 8 cM (500 markers total) intervals) and is used as the measure of genetic similarity at the marker. The IBD status at a marker is informative for the IBD status at any other locus on the chromosome as long as the population recombination fraction is less than 0.5. The IBD status at the marker and the locus are then correlated in the population and hence similarity at the marker is informative for similarity at the locus. The informative locus may be a gene or located near a gene. If variation in the gene and variation in the phenotype are related, then variation in the IBD status at the locus and thus also at the marker will be related to variation in phenotype similarity. It is possible to estimate the variance contributed by a genetic marker to a trait using structural equation modelling or other regression-based approaches.^39,40^

### 7.2 Candidate genes

Ultimately, we aim to quantify the specific effects of genes in terms of their trait contributions from additivity and dominance. Linkage analysis may identify a narrow chromosomal region (i.e. quantitative trait locus) containing a gene influencing the trait. Genes with putative function (i.e. ‘candidate’ genes) can then be selected to test for their association with the trait. Known as association analysis, regression-based methods can be used to test whether variation within the gene is related to variation within the trait.

Traditional association studies such as case–control designs may provide spurious associations, the result of stratified samples.^41^ This can be addressed by family-based methods in which locus-phenotype associations are compared across genetically-related individuals. The total population variance can be decomposed into two orthogonal components: between-family association and within-family association. Within-family association is robust to population stratification (i.e. spurious association due to allele frequency differences between subpopulations). For family-based designs, non-independence of cases can be taken into account by using a variance components framework.

### 7.3 Genome-wide association

Technological advances have now made association analysis possible on a genome-wide (GWA) level. Single nucleotide polymorphisms (SNPs; single DNA base changes, e.g. AAGGTTA to ATGGTTA) are the preferred markers, with sets of up to 500 K SNPs on a single chip (e.g. affymetrix). The first successfully documented GWA reported the findings of The Wellcome Trust Case Control Consortium Study.^38^ This was a case–control design with 14,000 cases and 3,000 shared British controls. Seven complex human diseases, including bipolar disorder, coronary artery disease, hypertension and diabetes, were examined using 500 K SNP chips. Population stratification was found to be modest provided non-Europeans were excluded. The study identified 24 primary signals (*p* < 5 × 10^−7^), almost all of which were supported by prior findings and/or replication studies. A further 58 loci were identified with *p* values < 10^−5^.

Family studies are not preferred for GWA because it requires very large samples for sufficient statistical power. However, family studies offer several advantages: they enable investigation of parent of origin effects, as maternal and paternal genes can be dissociated; gene x environment interaction effects can be studied, for instance, by measuring affected twins with known environmental exposures; and they are free from population stratification bias because the test of association can be tested within families as well as between families.

Once causal genetic polymorphisms are identified the next step is to describe gene (and protein) functions and interactions. Such studies will most likely target gene expression, protein analysis and structure (e.g. microarray studies, model organism studies).

## 8 The future

With recent advances in molecular biology and genome-scanning techniques, innovative approaches involving the study of twins have much to offer in complementing molecular studies and helping to unravel how genes and the environment contribute to both normal and abnormal phenotypic variation. As Martin et al.^10^ have pointed out, usually before starting to look for quantitative trait loci (QTLs) for complex traits, it is worthwhile to show that there is a significant component of genetic variation present. The classical twin model still provides a powerful way of confirming the presence of a genetic effect. Furthermore, when it comes to carrying out linkage and association studies, phenotypic data from MZ and DZ twins and other family members can be analysed simultaneously to achieve a gain in power to detect QTLs.^42,43^

Although the identification of key genes for dental development in humans will represent a major step forward, merely identifying genes will not explain fully how various dental anomalies arise in individuals. This is where further exploration of epigenetic factors, using the MZ co-twin model, is likely to be a fruitful area for future study. Already researchers are beginning to study epigenetic biomarkers in an attempt to explain the reasons for observed differences between MZ twin pairs.^44^ At this stage the focus is on determining the extent of differences in global genomic DNA methylation levels and it is likely that more specific analyses will be developed in the not-too-distant future. Once these approaches aimed at the level of DNA are refined further, and our understanding of the nature of the epigenetic influences at a local tissue level improves, we should be able to provide a clearer picture of how genetic, epigenetic and environmental factors interact to influence human dental development. With this knowledge, we will be in a better position to develop new preventive and therapeutic approaches for the management of many of the common developmental problems affecting the human dentition.

## Disclosures

*Competing interests:* None declared.

*Funding:*: National Health and Medical Research Council of Australia, Australian Dental Research Foundation, Wellcome Trust, UK.

## Figures and Tables

**Table 1:** Contribution of genetic and environmental components to variation in selected dental features in Australian twins.

Dental trait	Best-fitting model	h^2^	95% CI
Tooth emergence (i_1_)	AE	94	91–96

Intercuspal distances (M^1^)			
MB–DB	AE	60	29–78
DB–DL	AE	65	49–77
DL–ML	E	–	–
ML–MB	CE	–	–

Crown diameters (I^1^)			
MD	AE	88	–
LL	AE	80	–

Crown diameters (C)			
MD	ADE	86^a^	–
LL	AE	85	–

Crown diameters (M^1^)			
MD	ACE	59	46–69
BL	ACE	61	51–71

Carabelli trait	AE	90	–

Arch dimensions			
Breadth	AE	82	61–91
Depth	AE	92	81–97

Occlusal traits			
Overbite	AE	53	28–71
Overjet	AE	28	2–50

i_1_: primary mandibular central incisor; M^1^: permanent maxillary first molar; I^1^: permanent maxillary central incisor; C: permanent maxillary canine; MB: mesiobuccal; DB: distobuccal; DL: distolingual; ML: mesiolingual; MD: mesiodistal; LL: labiolingual; BL: buccolingual; A: additive genetic variance; D: non-additive genetic variance; C: common environmental variance; E: unique environmental variance; h^2^: narrow-sense heritability (additive genetic variance), 95% CI = 95% confidence interval reported when available.

a broad-sense heritability (additive plus non-additive genetic variance).
